# Intrinsic tryptophan fluorescence spectroscopy reliably determines galectin-ligand interactions

**DOI:** 10.1038/s41598-019-47658-8

**Published:** 2019-08-14

**Authors:** Paulina Sindrewicz, Xiaoxin Li, Edwin A. Yates, Jeremy E. Turnbull, Lu-Yun Lian, Lu-Gang Yu

**Affiliations:** 1 Department of Cellular and Molecular Physiology, Institute of translational Medicine, University of Liverpool, Liverpool, L69 3GE UK; 2 Department of Biochemistry, Institute of Integrative Biology, University of Liverpool, Liverpool, L69 7ZB UK

**Keywords:** Cancer screening, High-throughput screening

## Abstract

Galectins are involved in the regulation of divergent physiological and pathological processes and are increasingly recognized to play important roles in a number of diseases. However, a simple and effective way in assessing galectin-ligand interactions is lacking. Our examination of the sequence of all 12 human galectin members reveals the presence of one or more tryptophan residues in the carbohydrate-recognition domains of each galectin. This led us to investigate the possibility that alteration of the galectin intrinsic tryptophan fluorescence could be used in determining the strength of galectin-ligand interactions. One representative member from each of the three subtype galectins, galectin-2 (proto-), galectin-3 (chimera-) and galectin-4 (tandem repeat-type), was selected and analysed for galectin interaction with three ligands of different affinities: galactose, lactose and N-acetyl-lactosamine using tryptophan fluorescence spectroscopy (TFS) and, as a comparison, isothermal titration calorimetry (ITC). Good agreement between TFS and ITC measurements were revealed in ligand bindings of all galectin members. Moreover, TFS detected very weak galectin binding where ITC could not reliably do so. The reliability of TFS in determining galectin-ligand interactions was further validated by analysis of galectin-3 interaction with a semisynthetic ligand, F3. Thus, TFS can be used as a simple, sensitive and reliable way to determine galectin-ligand interactions and also as a drug-discovery platform in developing galectin-targeted therapeutic drugs.

## Introduction

Galectins are a family of 15 galactoside-binding mammalian proteins (12 in human). Each galectin contains one or two highly conserved carbohydrate-recognition domains (CRD) with a significant sequence similarity^1^. Galectins are divided into three subgroups based on their structural architecture. The prototype galectins in human include galectin-1, -2, -7, -10, -13, -14 and -16, each of which consists of one CRD and has the ability to form non-covalent homodimers with the exception of galectin-13 which dimerizes via disulphide bonds^2^. Galectin-3 is the exclusive chimera-type human galectin and possesses an unstructured N-terminal domain, rich in glycine, proline and tyrosine residues, that is fused to a single CRD. The tandem repeat-type human galectins include galectin-4, -8, -9 and -12 and are characterised by the existence of two CRDs within the same peptide chain connected by a short linker region^3^.

**Figure 1:**
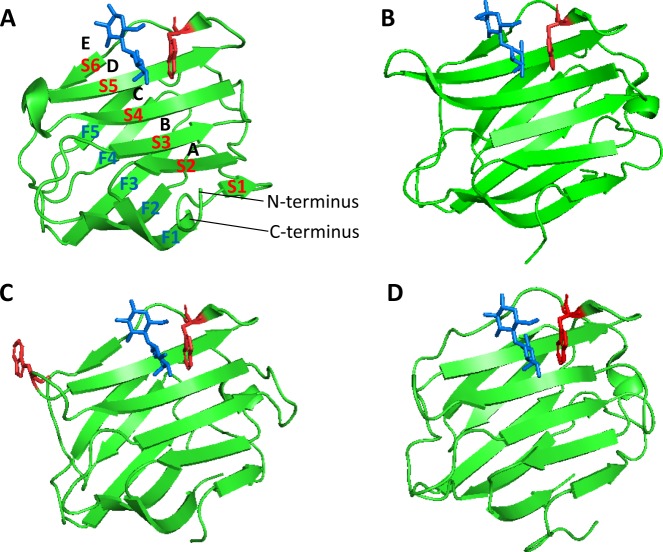
The CRDs of galectins-2, -3 and -4 and tryptophan residues in CRDs. The CRDs of galectin-2 (PDB code: 5DG2) (**A**) galectin-3 (3ZSJ) (**B**) galectin-4N (5DUV) (**C**) and galectin-4C (4YM3) (**D**) are shown as cartoon representations with tryptophan residues (red) and lactose molecules (blue) in stick representations. Galectin-2, galectin-3 and galectin-4C CRDs each contains one tryptophan and galectin-4N contains two tryptophan residues. Galectin binding subsites (**A**–**E**) strands S1–S6 and F1–F6 in CRD are also indicated in (**A**).

The CRD of each galectin consists of approximately 130–135 amino acid residues and is arranged into a globular β-sandwich structure formed by two antiparallel β-sheets. Six strands (S1–S6) form the concave S-sheet with the carbohydrate binding site whereas another five strands (F1-F5) form the F-sheet convex site^4^ (Fig. 1). The carbohydrate binding site of each galectin is long enough to accommodate a linear tetrasaccharide and can be divided into four main subsites (A–D) and an additional, less recognised subsite (E)^5^. Subsite C is the defining β-galactoside binding site and D serves as the second contributing part of the galectins canonical disaccharide-binding site^4^. C and D subsites form the core of galectin CRD which composes of highly conserved amino acid sequence among galectin members^4^. The sequence extension of CRD beyond the C and D subsites greatly increases the binding affinity of galectins with carbohydrates, especially with larger glycans. For example, poly-N-acetyllactosamine neo-glycoconjugates, which can interact with subsites A and B in addition to the core C and D subsites, have a much higher binding affinity with galectins than N-acetyl-D-lactosamine (LacNAc) which forms interactions with residues only in subsites C and D^1,6^.

Over the past decade, a huge body of evidence has revealed that galectins are multifunctional molecules and are involved in divergent biological activities in cell-cell and cell-environment communications^7–9^. They are increasingly recognized to play very important roles in a number of pathological processes such as inflammation, fibrosis, heart failure, diabetes and cancer^7,10^ For example, galectin-1 acts as an immunosuppressive agent in recovering immune cell homeostasis in autoimmunity and inflammation^9,11^, whereas galectin-3 plays a crucial role in cardiac fibrosis and remodelling, which contribute to the development and progression of heart failure^10,12^. Several galectins (e.g. galectin-1, -2, -3, -4, -7, -8) are active promotors in cancer development and progression by interaction with a range of galactose-terminated glycans^13,14^. With the increased realization of the functional importance of galectins in diseases, particularly in cancer and tissue fibrosis, significant attention has been drawn recently in academia and industry to develop galectin-targeted therapeutic agents^15^. A number of methods have been reported in assessing galectin-ligand interactions. However, all those methods rely heavily on either pre-conjugation of galectins/ligands^16–18^, or complex equipment and analysis systems^19,20^ and have limited detection sensitivity.

**Figure 2:**
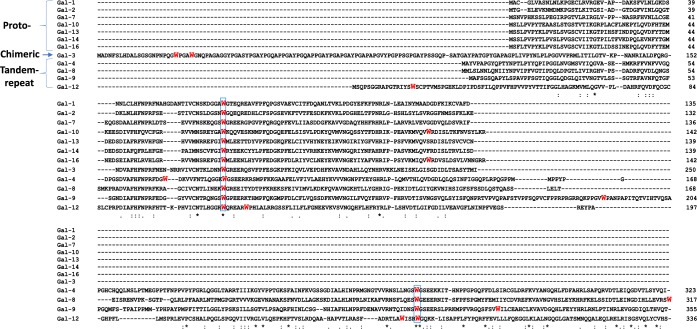
Multiple sequence alignment of members of human galectins. All the 12 known human galectins (Gal) are grouped on subtypes of prototypes: galectin-1 (UniProt code: P09382), galectin-2 (P05162), galectin-7 (P47929), galectin-10 (Q05315), galectin-13 (Q9UHV8), galectin-14 (Q8TCE9-1) and galectin-16 (A8MUM7); chimera type: galectin-3 (P17931) and tandem-repeat type: galectin-4 (P56470), galectin-8 (O00214-1), galectin-9 (O00182-1) and galectin-12 (Q96DT0-1). All tryptophan residues are coloured in red. Below the galectin sequence alignment is a key denoting conserved amino acid residues (*), conservative substitutions (:) and semi-conservative substitutions (.) within the Carbohydrate Recognition Domains (CRDs). The ubiquitous tryptophan residues in N- and C-terminal CRDs of all the galectin members are highlighted in blue brackets.

Tryptophan is known to emit intrinsic fluorescence that is measurable by fluorescence spectroscopy^21^. If the tryptophan residue of a protein is involved in interaction of the protein with a binding ligand, any change of the tryptophan microenvironment alters the tryptophan fluorescence spectrum and can cause a shift of the maximum fluorescence peak as well as variation of the fluorescence intensity^22,23^. Our examination of the sequences of all 12 human galectin members reveals the presence of at least one tryptophan residue in the CRD of each galectin (Figs 1 and 2). In addition to the key tryptophan residue in the CRDs (highlighted in blue brackets in Fig. 2), prototypic galectins-10 (Trp127) and -16 (Trp127) each contain an additional tryptophan residue in their CRDs whereas chimeric galectin-3 has two additional tryptophans (Trp22 and Trp26) in its N-terminal domain. The tandem-repeat type galectins-4, -8, -9 and -12 contain at least one tryptophan residue in each of their two CRD domains. Galectin-4 has two tryptophan residues (Trp71 and Trp84) in its N-terminal CRD, galectin-8 contains two (Trp 249 and Trp317) in the C-terminal domain whilst galectin-9 has two (Trp287, Trp309) in its C-terminal CRD and one (Trp185) in the linker region. Galectin-12 contains four tryptophan residues in its two CRDs, two (Trp117 and Trp123) in the N-terminal domain and two (Trp265 and Trp269) in its C-terminal domain.

The ubiquitous presence of tryptophan residues in the galectin CRDs led us to hypothesize that changes of the galectin intrinsic tryptophan fluorescence, upon ligand binding, might effectively reflect the strength of the galectin-ligand interactions. To test this hypothesis, we selected one member from each of the three subtype galectins, galectin-2 (prototypic), -3 (chimeric) and -4 (tandem repeat), and investigated their interactions with three known galectin ligands of different binding affinities, galactose, lactose and LacNAc by tryptophan fluorescence spectroscopy (TFS) and, as a comparison, also by the classical method for determining protein-ligand interactions, isothermal titration calorimetry (ITC).

## Results

### Galectin-3-ligand interactions determined by TFS and ITC

**Figure 3:**
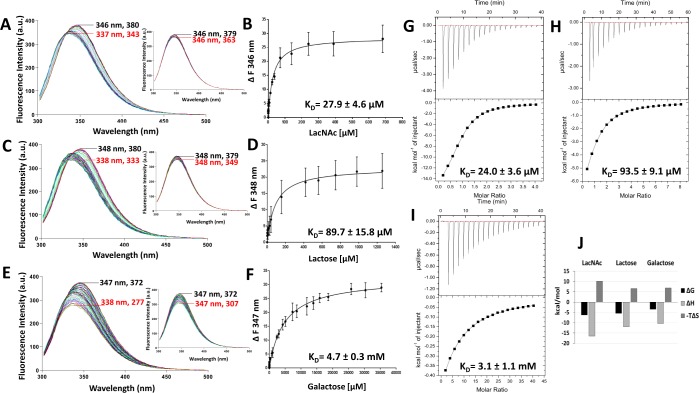
Determination of galectin-3-ligand interactions by TFS and ITC. Galectin-3 (10 µM) florescence intensity with increased concentrations of LacNAc (**A**) lactose (**C**) and galactose (**E**) were recorded by fluorescence spectroscopy. Numbers indicate the maximum fluorescence wavelength and corresponding fluorescence intensity for the first (no ligand/buffer titration, black) and last (red) titration traces. Inserts in each panel show buffer control titrations. Ligand concentrations vs changes in fluorescence intensity at single wavelength were plotted and analysed using *One site- Specific binding* model to obtain the galectin-3 binding affinities to LacNAc (**B**) lactose (**D**) and galactose (**F**). Heat changes for titration of galectin-3 (100 µM) with 2 mM LacNAc (**G**), 4 mM lactose (**H**) and 20 mM galactose (**I**) were recorded by ITC (upper panels) with data fit to *One set of sites* interaction model (bottom panels). Data were plotted as means ± SEM from three independent experiments for each assessment. Panel (J) shows thermodynamic profiles with enthalpy (∆H), entropy (∆S) and Gibbs free energy (∆G) changes associated with galectin-3 binding to LacNAc, lactose and galactose.

Galectin-3, the only chimera type galectin, contains three tryptophan residues; one in the CRD (Trp181), which is well conserved among all human galectins and is directly involved in galectin carbohydrate binding (Figs 1 and 2), and two in the N-terminal domain (Trp22, Trp26) (Fig. 2). The maximum tryptophan fluorescence of galectin-3 occurred between 346 and 348 nm (Fig. 3A). Titration of galectin-3 with LacNAc (Fig. 3A), lactose (Fig. 3C) or galactose (Fig. 3E) resulted in shifts (9–10 nm) of the galectin-3 fluorescence emission maxima to lower wavelengths (blue shift) in a ligand concentration-dependent manner (Fig. 3A,C,E). Such shifts of fluorescence peaks, known to result from the Trp becoming more buried and shielded away from the hydrophilic environment^24^, are indication of changes to the local environment around the Trp residue. Introduction of the galectin-3 ligands also reduced the galectin-3 tryptophan fluorescence intensity in a ligand concentration-dependent manner. The background fluorescence quenching caused by protein dilution with the buffer was monitored by running parallel buffer control titrations. Buffer titration resulted only in reduction of fluorescence intensity but no shifts of the maximum fluorescence peaks (Fig. 3A,C,E, inserts). Once the changes of buffer control titrations ran in parallel were subtracted, the maximum ligand binding- induced changes of the intrinsic fluorescence intensity were 26, 21 and 28 units for LacNAc, lactose and galactose, respectively. Changes of the tryptophan fluorescence in response to titration of the binding ligands vs ligand concentration were plotted (Fig. 3B,D,F) and the binding affinities (K_D_) for each ligand were determined. As expected, LacNAc emerged as the strongest galectin-3 binding ligand (K_D_, 27.9 ± 4.6 µM), followed by lactose (K_D_, 89.7 ± 15.8 µM) and galactose (K_D_, 4.7 ± 0.3 mM) (Fig. 3B,D,F). The presence of lactose showed no effect at all on the maximum tryptophan fluorescence of BSA (bovine serum albumin) (345 nm), a non-glycan binding and three tryptophan residue-containing protein, and, as expected, caused only a small reduction of fluorescence intensity due to buffer dilution (Fig. S1). This provides further support to the effectiveness of TFS analysis in determining galectin-3-ligand interactions.

To compare the binding characteristics obtained by TFS, galectin-3 interactions with these ligands were also assessed by ITC. The ITC titration traces fitted well into a single-site binding model for each binding ligand (Fig. 3G–I). LacNAc showed ~4-fold stronger binding to galectin-3 (K_D_, 24.0 ± 3.6 µM) (Fig. 3G) than lactose (K_D_, 93.5 ± 9.1 µM) whereas galactose showed very weak binding (K_D_, 3.1 ± 1.1 mM). The K_D_ values of the binding of these ligands to galectin-3 revealed here by ITC are similar to those reported previously for galectin-3 binding to those ligands obtained by other methods^17,25–27^ and are in good agreement with the dissociation constants obtained by TFS (Fig. 3B,D,F).

Further analyses of the thermodynamics of galectin-3 binding by these ligands carried out reveal that LacNAc binding to galectin-3 is enthalpically-driven, with a high negative enthalpy, ∆H, of -16.5 kcal/mol (Fig. 3J). This is consistent with previous reports of the involvement of hydrogen bonding and Van der Waals interactions in galectin-3-LacNAc interactions^28^. The galectin-3 interactions with lactose and galactose showed similar binding thermodynamic characteristics of favourable binding enthalpy (∆H) and entropy factor (T∆S) as with LacNAc (Fig. 3J). A galactose residue is the minimal carbohydrate binding moiety for galectin-3 and showed the least number of interactions with less favourable binding enthalpy (∆H) and entropy factors (T∆S) than lactose and LacNAc, both of which have a second glucose-based binding moiety and form more interactions with galectin-3. There is a difference of 6.1 kcal/mol in the binding enthalpy from the weakest ligand galactose to the strongest binding ligand LacNAc in galectin-3 binding. The difference in enthalpy change of 4.4 kcal/mol between LacNAc and lactose can be attributed to the N-acetyl group of the glucose moiety in LacNAc^29,30^ which forms Van der Waals interactions between the methyl group and Arg186 of galectin-3 and also forms water-mediated hydrogen bond between the amide proton and Glu-165^30^.

### Galectin-2-ligand interactions determined by TFS and ITC

**Figure 4:**
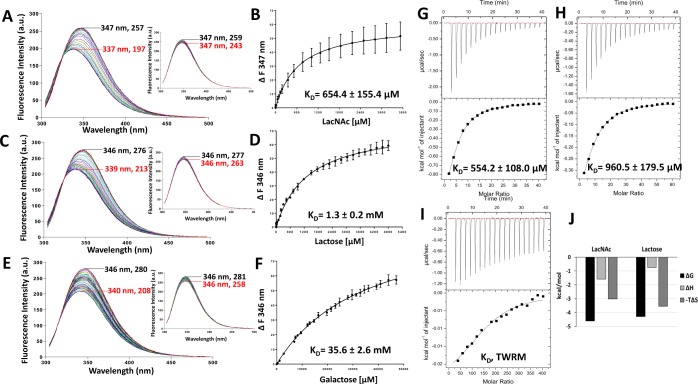
Determination of galectin-2-ligand interaction by TFS and ITC. Galectin-2 (20 µM) florescence intensity with increased concentrations of LacNAc (**A**), lactose (**C**) and galactose (**E**) were recorded by fluorescence spectroscopy. Numbers indicate the maximum fluorescence wavelength and corresponding fluorescence intensity for the first (no ligand/buffer titration, black) and last (red) titration traces. Inserts in each figure show buffer control titrations. Ligand concentrations vs changes in fluorescence intensity were plotted and analysed using *One site- Specific binding* model to obtain the binding affinities to LacNAc (**B**), lactose (**D**) and galactose (**F**). Heat changes for titration of galectin-2 (100 µM) with 20 mM LacNAc (**G**), 30 mM lactose (**H**) and 200 mM galactose (**I**) were measured by ITC (upper panels) with data fitting to *One set of sites* interaction model (bottom panels). Data were plotted as means ± SEM from three independent experiments for each assessment. (TWRD, too weak to be reliably determined). Panel (J) shows thermodynamic profiles with enthalpy (∆H), entropy (∆S) and Gibbs free energy (∆G) changes associated with galectin-2 binding to LacNAc and lactose.

A similar strategy of a combined use of TFS and ITC measurements described for galectin-3 was next adopted to characterise galectin-2 binding to the same ligands. Galectin-2 is one of seven human proto-type galectins. Like the other proto-type galectins (Figs 1 and 2), galectin-2 contains one tryptophan residue (Trp65) in its CRD which is directly involved in glycan binding^30,31^. The maximum of tryptophan fluorescence emission peak of galectin-2 occurred between 346 and 347 nm (Fig. 4A,C,E). Titration of increasing amounts of LacNAc, lactose and galactose, resulted in blue shifts of 10, 7 and 6 nm, respectively, of the galectin-2 maximum fluorescence peaks to a lower wavelength in a ligand concentration-dependent way. Similarly, the intensity of the tryptophan fluorescence signal was quenched with increasing concentrations of the ligand. After subtraction of the changes induced by buffer titrations, the maximum ligand binding-induced fluorescence intensity changes were shown to be 52, 59 and 57 units for LacNAc, lactose and galactose, respectively. The K_D_ values of LacNAc, lactose and galactose binding to galectin-2 were 654.4 ± 155.4 µM, 1.3 ± 0.2 mM and 35.6 ± 2.6 mM, respectively (Fig. 4B,D,F).

The titration traces of these ligands to galectin-2 in ITC fitted well into *One site interaction* model for each binding ligand (Fig. 4G–I). The binding affinity of LacNAc to galectin-2 (K_D_, 554.2 ± 108.0 µM) was ~2-fold stronger than for lactose (K_D_, 960.5 ± 179.5 µM). Interestingly, the binding of these two ligands to galectin-2 have very favourable entropic energies (Fig. 4J). Galactose binding to galectin-2 was too weak to be reliably determined by ITC (Fig. 4I). The binding affinities of LacNAc and lactose revealed by ITC are in excellent agreement with that obtained by TFS (Fig. 4A–F). A previous study using frontal affinity chromatography reported the binding affinity of lactose to galectin-2 to be 1.1 mM^20^ which was similar to that obtained by TFS and ITC in this study.

### Galectin-4-ligand interactions determined by TFS and ITC

**Figure 5:**
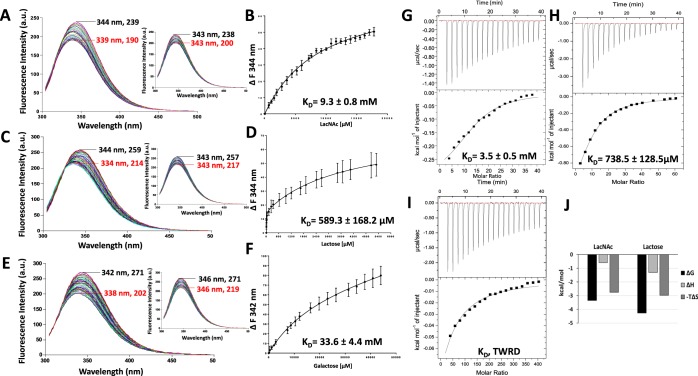
Determination of galectin-4-ligand interactions by TFS and ITC. Galectin-4 (5 µM) florescence intensity with increased concentrations of LacNAc (**A**), lactose (**C**) and galactose (**E**) were recorded by fluorescence spectroscopy. Numbers indicate the maximum fluorescence wavelength and corresponding fluorescence intensity for the first (no ligand/buffer titration, black) and last (red) titration traces. Inserts in each figure show buffer control titrations. Ligand concentrations vs changes in fluorescence intensity were plotted and analysed using *One site- Specific binding* model to obtain the binding affinities to LacNAc (**B**), lactose (**D**) and galactose (**F**). Heat changes for titration of galectin-4 (100 µM) with 10 mM LacNAc (**G**), 30 mM lactose (**H**) and 200 mM galactose (**I**) were measured by ITC (upper panels) with data fit to *One set of sites* interaction model (bottom panels). Data were plotted as means ± SEM from three independent experiments for each assessment. Panel (J) shows thermodynamic profiles with enthalpy (∆H), entropy (∆S) and Gibbs free energy (∆G) changes associated with galectin-4 binding to LacNAc and lactose.

TFS and ITC analysis were also applied to binding of these ligands to galectin-4, an example of the tandem repeat-type of galectins. Galectin-4 contains three tryptophan residues in its two separate CRDs, two in the N-terminal CRD (Trp71 and Trp84) and one in the C-terminal CRD (Trp256) (Fig. 2). The fluorescence emission peak for galectin-4 occurred at 342–344 nm. Titration of LacNAc, lactose and galactose to galectin-4, as for galectin-2 and -3, resulted in both blue shifts of the maximum fluorescence peaks (by 5, 10 and 4 nm for LacNAc, lactose and galactose, respectively) to lower wavelengths as well as reduction of the galectin-4 tryptophan fluorescence intensity in a ligand concentration-dependent manner (Fig. 5A,C,E). After deduction of the changes of the control buffer titration, the maximum ligand binding-induced fluorescence intensity changes for galectin-4 were 50, 49 and 80 units for LacNAc, lactose and galactose, respectively (Fig. 5A–F). The binding of galectin-4 to lactose (K_D_, 589.3 ± 168.2 µM) was stronger than to LacNAc (K_D_, 9.3 ± 0.8 mM). Again, galactose binding was very weak (K_D_, 33.6 ± 4.4 mM).

ITC analysis of the galectin-4 binding revealed very similar results to that obtained by TFS, in which the binding affinity for lactose (K_D_, 738.5 ± 128.5 µM) being greater than for LacNAc (K_D_ 3.5 ± 0.5 mM) (Fig. 5G,H). The thermodynamics data show that lactose and LacNAc binding are driven by favourable entropic and enthalpic energies (Fig. 5J).

Galactose binding to galectin-4 was too weak to be reliably determined by ITC (Fig. 5I). The binding affinity of galectin-4 to lactose obtained by TFS (589.3 µM) and ITC (738.5 µM) are similar to that obtained by Surface Plasmon Resonance (SPR) analysis (860 µM) in an earlier study^32^.

**Figure 6:**
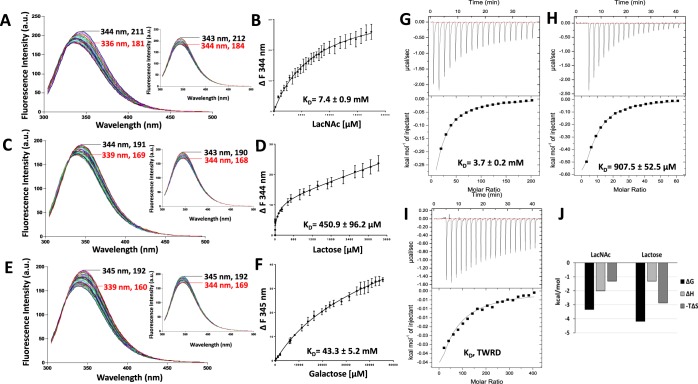
Determination of galectin-4N-ligand interactions by TFS and ITC. Galectin-4N (5 µM) florescence intensity with increased concentrations of LacNAc (**A**), lactose (**C**) and galactose (**E**) were recorded by fluorescence spectroscopy. Numbers indicate the maximum fluorescence wavelength and corresponding fluorescence intensity for the first (no ligand/buffer titration, black) and last (red) titration traces. Inserts in each figure show buffer control titrations. Ligand concentrations vs changes in fluorescence intensity were plotted and analysed using *One site- Specific binding* model to obtain the binding affinities to LacNAc (**B**), lactose (**D**) and galactose (**F**). Heat changes for titration of galectin-4N (100 µM) with 20 mM LacNAc (**G**), 30 mM lactose (**H**) and 200 mM galactose (**I**) were measured by ITC (upper panels) with data fit to *One set of sites* interaction model (bottom panels). Note the very small heat change by galactose which indicates very little, if any, binding taking place (**I**). Data were plotted as means ± SEM from three independent experiments for each assessment. Panel (J) shows thermodynamic profiles with enthalpy (∆H), entropy (∆S) and Gibbs free energy (∆G) changes associated with galectin-4N binding to LacNAc and lactose.

**Figure 7:**
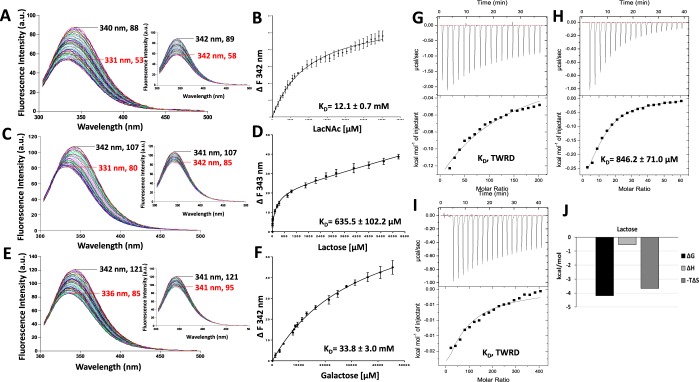
Determination of galectin-4C-ligand interactions by TFS and ITC. Galectin-4C (5 µM) florescence intensity with increased concentrations of LacNAc (**A**), lactose (**C**) and galactose (**E**) were recorded by fluorescence spectroscopy. Numbers indicate the maximum fluorescence wavelength and corresponding fluorescence intensity for the first (no ligand/buffer titration, black) and last (red) titration traces. Inserts in each figure show buffer control titrations. Ligand concentrations vs changes in fluorescence intensity were plotted and analysed using *One site- Specific binding* model to obtain the binding affinities to LacNAc (**B**), lactose (**D**) and galactose (**F**). Heat changes for titration of galectin-4C (100 µM) with 20 mM LacNAc (**G**), 30 mM lactose (**H**) and 200 mM galactose (**I**) were measured by ITC (upper panels) with data fit to *One set of sites* interaction model (bottom panels). Note the very small heat change by LacNAc (**G**) and galactose (**I**) which indicates very little, if any, binding taking place. Data were plotted as means ± SEM from three independent experiments for each assessment. Panel (J) shows thermodynamic profiles with enthalpy (∆H), entropy (∆S) and Gibbs free energy (∆G) changes associated with galectin-4C binding to lactose.

In an attempt to understand in more details the binding characteristics of the two CRD domains of galectin-4, the two galectin-4 CRDs were expressed and produced as separate domains, termed galectin-4N (residues 1–152) and galectin-4C (residues 153–323). The galectin-4N tryptophan fluorescence emission peak occurred at wavelength 343–345 nm (Fig. 6A,C,E) whereas galectin-4C occurred at slightly lower wavelength at 340–342 nm (Fig. 7A,C,E). The basal tryptophan fluorescence intensity of galectin-4C, -4N and -4full length was 88~89, 211~212, and 238~239 a.u., respectively, the increase of intensity was correlated closely with the increased number of tryptophan residues in galectin-4C, -4N and -4full length (one, two and three residues, respectively). Similar to the full-length galectin-4, titration of each of the binding ligands into the protein resulted in blue shifts of the maximum fluorescence intensity peaks to lower wavelengths as well as reduction of the fluorescence intensity of galectin-4N and galectin-4C in a ligand concentration-dependent manner (Fig. 6A,C,E for galectin-4N and Fig. 7A,C,E for galectin-4C). As for full-length galectin-4, lactose binding to both galectin-4N (K_D_, 450.9 ± 96.2 µM) and –4 C (K_D_, 635.5 ± 102.2 µM) was stronger than LacNAc bindings (K_D_, 7.4 ± 0.9 mM, 12.1 ± 0.7 mM, respectively). Galactose binding to galectin-4N and -4C were both extremely weak (Figs 6F and 7F).

The ITC data for the galectin-4 domains followed the same pattern as the TFS data, with lactose binding strongest to both galectin-4N (K_D_, 907.5 ± 52.5 µM) and galectin-4C (K_D_, 846.2 ± 71.0 µM) (Figs 6H and 7H) among the three ligands, and in both cases entropically-driven (Figs 6J and 7J). Binding of LacNAc to galectin-4N was weaker than to lactose with a Kd of 3.7 + 0.2 mM, again this being entropically driven (Fig. 6G,J). Binding of galactose to galectin-4N, and of both LacNAc and galactose to –galectin-4C were very too weak and mostly beyond detection by ITC (Figs 6I and 7G,I).

### Determination of galectin-3 interaction with a novel binding inhibitor from chemically modified heparin

**Figure 8:**
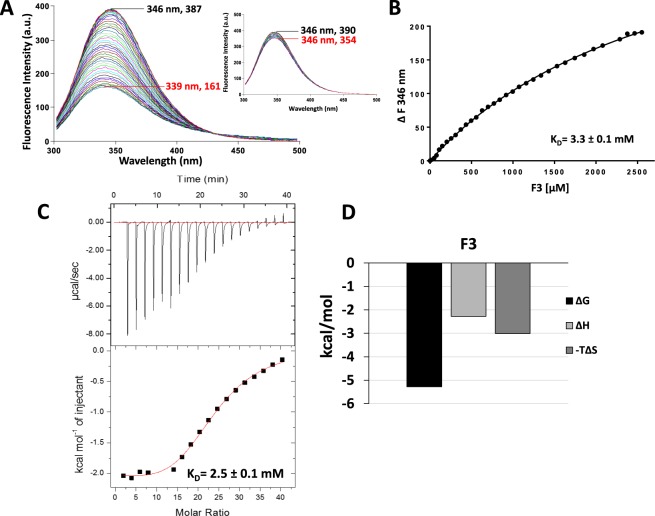
Analysis of galectin-3 interaction with ligand F3 by TFS and ITC. Galectin-3 (10 µM) florescence intensity with increased concentrations of F3 were recorded by fluorescence spectroscopy (**A**). Numbers indicate the maximum fluorescence wavelength and corresponding fluorescence intensity for the first (no F3/buffer titration, black) and last (red) titration traces. Insert in the figure shows buffer control titrations. Ligand concentrations vs changes in fluorescence intensity were plotted and analysed using nonlinear curve fitting with *One site- Specific binding* model and the galectin-3 binding affinity to F3 was determined (**B**). Heat changes for titration of galectin-3 (100 µM) with 20 mM F3 measured by ITC are shown in (**C**) (upper panel) with logistic fit of the data (bottom panel). Panel (D) shows thermodynamic profiles with enthalpy (∆H), entropy (∆S) and Gibbs free energy (∆G) changes associated with galectin-3 binding to F3.

The consistent agreement of bindings obtained by TFS and ITC for all the three subtypes of galectins indicates that measurements of intrinsic tryptophan fluorescence changes can serve as a reliable way to determine galectin-ligand interactions. To demonstrate this further, we assessed the interaction of galectin-3 with a new binding ligand F3, a small molecular weight (<3 kDa) sub-fraction of 6-de-O-sulfated, N-acetylated heparin recently identified in our labs from chemically modified heparins^33^. Our previous studies showed that F3 binds to the galectin-3 CRD, induces galectin-3 conformational changes and inhibits galectin-3-mediated cancer cell adhesion and metastasis^33^. In this study, the presence of F3 caused substantial reduction of galectin-3 intrinsic fluorescence intensity (190 units) and a shift (7 nm) of the maximum fluorescence peak to lower wavelength (Fig. 8A). The dissociation constant of F3 binding to galectin-3 was determined to be K_D_, 3.3 ± 0.1 mM (Fig. 8B). ITC analysis showed similar binding affinity (KD, 2.5 ± 0.1 mM) of F3 interaction with galectin-3 (Fig. 8C,D) as that determined by TFS. The excellent agreement of results for galectin-3-F3 interactions obtained by TFS and ITC provides further strong support for the effectiveness and reliability of measuring intrinsic galectin tryptophan fluorescence in determining galectin-ligand interactions. As F3 is a heparin derivative of complex glycan, these results also indicate that TFS analysis can be used in assessing galectin interactions with simple ligand as well as complex glycans.

## Discussion

The ubiquitous presence of tryptophan residues in the CRDs of all members of human galectins and the nature of intrinsic emission of fluorescence by tryptophan residues led us to test the hypothesis in this study that changes of galectin intrinsic tryptophan fluorescence in response to ligand binding might reliably reflect the strength of galectin-ligand interactions. It was found that the binding of three galectin binding ligands with different binding affinities to each example of the three subtype galectins galectin-2 (proto-type), galectin-3 (chimera-type) and galectin-4 (tandem repeat-type) can be quickly determined by intrinsic tryptophan fluorescence spectroscopy. The binding affinities obtained from TFS were in good agreement with that obtained by ITC, the “gold-standard” measurement in assessing protein-ligand interactions. The validity and effectiveness of the TFS measurement were further confirmed by analysis of galectin-3 interaction with a newly identified ligand F3.

Although galectin family members share high sequence and structural similarities, each galectin member is known to have unique binding specificity toward galactose-terminated glycans, largely due to differences in their ability to accommodate saccharides in the D, B and A subsites of their CRD regions^4,16^. In this study, TFS measurements demonstrated different binding affinities of LacNAc, lactose and galactose to each of the three exemplar galectins, with LacNAc being the strongest ligand for galectin-2 and -3 while galactose the weakest to all the three galectins. The differences in the relative binding affinities of these ligands for each of the galectins revealed by TFS were very consistent with that obtained by ITC. The agreement between TFS and ITC measurements for LacNAc, lactose and galactose binding are particularly strong for galectin-2 and -3. Galectin-2, like all the other prototype galectins-1, -7, -10, -13, -14 and -16, contains a single tryptophan residue located in its carbohydrate binding site. The excellent agreement of galectin-2 binding to each of the three ligands between TFS and ITC indicates that TFS is readily applicable to determine ligand interactions of all prototype galectins, in addition to the chimera-type galectin-3.

Galectin-4 binding by LacNAc, lactose and galactose also showed very good agreement between TFS and ITC measurements. Unlike galectin-2 and -3 where LacNAc binding is stronger than lactose, the reverse appears to be the case for galectin-4 as well as for its two separate CRDs. The poorer binding of LacNAc to galectin-4 compared to lactose is in agreement with other studies. LacNAc binding to galectin-4C was shown to be at least 11-fold weaker than lactose in early reports analysed by fluorescence anisotropy^16,34^. It was believed that the enhanced binding affinity of LacNAc to most other galectin members such as galectin-3 is attributed to the N-acetyl interaction with a conserved arginine residue in the CRDs of most galectin members. This arginine residue is, however, missing from the C-terminal CRD of galectin-4^34^.

In this study, TFS and ITC analyses showed little difference in binding of the three forms of galectin-4, the full-length, N- and C-terminal CRDs, to each of the individual ligand of LacNAc, lactose and galactose. For example, lactose binds to the full-length galectin-4 with K_D_ ~589 µM, to galectin-4N K_D_ ~450 µM and galectin-4C K_D_ ~635 µM. The N-terminal domain of galectin-4 contains two tryptophan residues (Trp71 and Trp84), one (Trp71) of which is distant from the carbohydrate binding site, whilst the C-terminal CRD contains one tryptophan residue (Figs 1 and 2). The discovery that binding to galectin-4N or -4C by each of the three ligands is similar indicates minimal influence of the distant tryptophan residue in galectin-4N toward galactin-4-ligand interactions. It should be mentioned, however, that the lack of difference between galectin-4N and -4C in ligand binding may be limited to only short chain oligosaccharides as galectin-4N has been reported to bind to more complex sulfated saccharides with higher specificity than galectin-4C^35^.

The binding of galactose to each of these galectins is very weak, from K_D_ ~5 mM (to galectin-3) to K_D_ 30~40 mM (to galectin-2 and -4) by TFS and mostly (to galectin-2 and -4) too weak to be determined by ITC where the threshold in reliably quantifying interactions is recognized to be K_D_ at approximately 1 mM^19^. Although using unconventionally high concentrations of galectins and ligands, we were able to detect measurable ligand binding with K_D_ slightly higher than 1 mM in this study (e.g. galactose to galectin-3), reliably quantifying binding of exceptionally poor ligands (e.g. galactose binding to galectin-2 and -4) using ITC remains a challenge. Often, for poor binders, the ligand binding induced-heat changes are so small that are almost comparable to the heat changes induced by control buffer dilution. Given that the extremely weak binding of galactose to galectin-2 and -4 were readily detected by measuring changes of intrinsic tryptophan fluorescence, TFS may have a particular advantage over the ITC for detecting galectin interactions with weak binding ligands. It is worth noting, however, that in cases where the ITC experiments are able to yield reliable results, the thermodynamics profile (ΔH and -TΔS) are useful in distinguishing between direct hydrogen bonds formed directly between the protein and ligand (enthalpically-driven interactions) from water-bridged hydrogen bonds (entropically-driven interactions).

In addition to ITC, several other methods have been reported previously in studying glycan interactions with limited galectin members. Fluorescence anisotropy, in which saccharides were first labelled with fluorescein tags, was used to investigate interactions of modified glycans with galectin-1, galectin-3 and galectin-4^16,36^. Tryptophan fluorescence anisotropy was used in investigation of the interaction of wheat germ agglutinin with LacNAc and also of galectin-1 with lactose^37–39^. Biolayer Interferometry has been used to determine binding of immobilised biotinylated galectin-1, -3 and -7 with LacNAc^17^ and SPR were used to determine interaction of galectin-1, -2 and -3 with biotin-conjugated glycosides coupled to a sensor surface^18^. Those methods, however, mostly rely heavily on pre-conjugation of galectins (e.g. Biolayer Interferometry) or tags of the binding ligands with biotin or fluorescence (e.g. fluorescence anisotropy and Surface Plasmon Resonance). Conjugation of galectins or ligands with tags may alter the natural conformation of galectins and/or ligands and consequently, may affect the accuracy of the galectin-ligand interaction measurements. Tryptophan fluorescence spectroscopy has been used in studying the unfolding process of porcine galectin-1 in the presence of a chemical denaturing agent GdnHCl and in analysing interaction of human galectin-1 with galactodendritic porphyrin^40^.

The label-free TFS measurements shown in this study have a number of advantages over the other methods used in assessing galectin-ligand interactions. As an internal binding sensor in the galectin CRD, tryptophan fluorescence is very sensitive to changes in polarity of its local environment and thus provides good sensitivity to determine ligand binding as shown in detection of the weak ligand galactose to galectin-2 and -4 binding in this study. In addition to its ability to detect weak galectin-ligand interactions, TFS analysis is simple and does not require any pre-conjugation of either galectins or ligands. In comparison to the other methods such as ITC, which typically requires >100 µM galectins to obtain a good titration trace^16,25^, TFS analysis requires much less galectin (5~10 µM). The requirement of lower galectin concentration in TFS analysis is particularly advantageous since many galectin members have tendencies to form aggregates and precipitates at higher concentrations. Since the measurement does not require pre-conjugation of galectins or ligands, TFS analysis is quick and takes about 30–60 minutes including running parallel buffer control titration. It also requires much less expensive equipment (only a standard spectrofluorimeter) than most of the other methods. This makes the TFS method very attractive as a screening platform in drug discovery to identify novel galectin binding inhibitors from compound libraries. The revelation that interaction of galectin-3 with the ~3 kDa inhibitor F3 could be quickly and accurately determined by TFS indicates that this method can be used to determine galectin interactions with small as well as large binding ligands. Moreover, TFS, like ITC, can be carried out at different temperatures to suit different biological conditions. Perhaps the biggest advantage of TFS analysis in comparison to the other methods is its readily application to all members of the human galectins, hence making this method attractive for rapid screening of potential galectin inhibitors.

In conclusion, TFS can be used as a simple and reliable way to determine interaction of human galectins with large and small ligands of high and low binding affinities. It can be used broadly in studying galectin-ligand interactions in various biological and biophysical systems. It also provides a very useful screening platform to identify novel galectin-binding inhibitors for development of galectin-targeted therapeutic drugs.

## Experimental Procedures

### Materials

N-Acetyl-D-lactosamine was from Dextra Laboratories Ltd (Reading, UK), α-lactose was from Acros Organics (Loughborough, UK). The expression vector pOPINS was from Oxford Protein Production Facility UK (Harwell Oxford, UK), pETM-11 from European Molecular Biology Laboratory (Heidelberg, Germany) and pET47b from Novagen Merck Millipore (Watford, UK). Chemically competent bacterial *E*. *coli* BL21(DE3) cells were purchased from New England Biolabs (NEB) (Hitchin, UK), LB media and LB agar were obtained from Merck Millipore (Darmstadt, Germany) and Kanamycin Sulfate from Melford (Chelsworth, UK). Isopropyl β-D-1-thiogalactopyranoside (IPTG) was from Apollo Scientific (Stockport, UK), DNAse, D-galactose and Lysozyme from hen egg from Sigma-Aldrich (Dorset, UK) and complete EDTA-free cocktail protease inhibitor tablets from Roche (Mannheim, Germany). All HisTrap FF and Superdex 75 26/60 columns that were used for galectin purifications were purchased from GE Healthcare (Little Chalfont, UK).

### Plasmid construction for recombinant human galectin-2, -3, -4, -4N and -4C

Codon-optimised synthetic genes were used to clone the expression constructs. Sequences encoding full length human galectin-2 (residues 1–132, UniProt code: P05162) and full-length human galectin-4 (residues 1–323, UniProt code: P56470) were cloned into pOPINS expression vector with a 6xHis-SUMO-tag at the N-terminus. The cDNA sequence encoding full-length human galectin-3 (residues 1–250, UniProt code: P17931) was cloned into pETM-11 expression vector with a His-tag at the N-terminus. The sequence of N-terminal galectin-4 (galectin-4N, residues 1–152) and C-terminal galectin-4 (galectin-4C, residues 153–323) were amplified from the same cDNA sequence encoding galectin-4 by PCR and cloned into pET47b expression vectors. The vectors were analysed and correct sequences were confirmed by either SUPREMERUN Sanger sequencing (Eurofins Genomics, Ebersberg, Germany) (galectin-2 and galectin-4) or Sanger DNA sequencing (Deep Seq, University of Nottingham, Nottingham, UK) (galectin-3).

### Production of recombinant human galectins

Competent *E*. *coli* BL21(DE3) cells were transformed by addition of 50–100 ng recombinant plasmid, heated at 42 °C for 45 seconds in a heat block and followed by 1 hr recovery in LB media at 37 °C with shaking (250 rpm). The transformed cells were selected on LB agar plates containing 50 µg/ml kanamycin. Galectins expression was induced with 0.5 mM IPTG when the cell density (OD_600_) reached approximately 0.6–0.85. After overnight incubation at 18 °C with shaking (200 rpm), the cells were harvested by centrifugation at 5,000 g for 30 min. Following incubation with 10 µg/ml DNAase in HisTrap buffer A (50 mM Tris 500 mM NaCl 20 mM Imidazole pH 7.5) containing EDTA-free protein inhibitor cocktail and 1 mg/ml lysozyme, the cell suspension was passed through a cell homogeniser. After centrifugation at 20,000 g for 1 hr, the supernatant was collected and applied onto a HisTrap FF 5 ml column and the His-tagged proteins were eluted with 150 mM Imidazole. All collected fractions were dialysed overnight, with the appropriate proteases, in a 3.0 kDa MWCO dialysis tubing against HisTrap buffer A: galectin-2 with SUMO protease (1 mg of SUMO protease per 50 mg of target protein), galectin-3 with TEV protease (in 1:20 TEV protease:protein ratio), galectin-4 with Sumo protease (1 mg of SUMO protease per 50 mg of target protein), and galectin-4N and -4C with HRV3C protease (1 mg of HRV3C protease per 25 mg of target protein). After performing Reverse HisTrap chromatography to remove the cleaved tags and proteases, each galectin solution was applied to Superdex 75 26/60 column to obtain purified recombinant galectins. Size-exclusion chromatography followed by multiangle light scattering (SEC-MALS) showed that all the proteins used for the TFS and ITC experiments are monomeric for galectin-2, galectin-3 and also predominantly for galectin-4. The purity of the recombinant galectins was determined by SDS-PAGE to be >95%. The functions of the generated recombinant galectins were confirmed by their binding to asialofetuin by ELISA and by aggregation of human red blood cells.

### Production of galectin-3 inhibitor, F3

The galectin-3 binding inhibitor, F3, was produced from chemically modified heparins and comprises a low molecular weight (<3 KDa) fraction of 6-de-O-sulfated, N-acetylated heparin as described previously^33^.

### Assessments of galectin-ligand interactions by TFS

All tryptophan fluorescence spectroscopy experiments were performed using CARY Eclipse Fluorescence Spectrophotometer (Varian Inc. Santa Clara, California, USA) and a 3 ml quartz cuvette. The excitation wavelength was fixed to 285 nm (little spectra difference was observed at 285 and 295 nm but better signal:noise ratio was achieved at 285 nm) and emission spectra were collected between 300 and 500 nm with a slit width of 5 nm. The temperature was maintained constant at 25 °C by an external thermostatic water circulator. To measure galectin-ligand interactions, recombinant galectin-2 at 20 µM, galectin-3 at 10 µM and galectin-4, -4N and -4C at 5 µM were allowed to equilibrate in PBS for 10–30 minutes under constant stirring before being titrated with testing carbohydrate ligand solutions. Galectin-2 was tested with increasing concentrations of LacNAc from 0 to 3.5 mM, lactose from 0 to 4.8 mM and galactose from 0 to 47.0 mM. Galectin-3 was analysed with ligands ranging from 0 to 700 µM for LacNAc, 0 to 1.25 mM for lactose and 0 to 35.5 mM for galactose whereas the inhibitor F3 was tested at concentrations ranging from 0 to 2.64 mM. Galectin-4 was tested with 0 to 17.8 mM for LacNAc, 0 to 5.3 mM for lactose and 0 to 47.0 mM for galactose, galectin-4N from 0 to 17.4 mM for LacNAc, 0 to 3.8 mM for lactose and 0 to 47.0 mM for galactose whereas the concentrations used for galectin-4C were 0 to 36.2 mM for LacNAc, 0 to 6.3 mM for lactose and 0 to 47.0 mM for galactose. Control buffer titrations to galectin solutions were performed in parallel for background determination in each experiment. Data from three independent experiments were analysed using nonlinear regression with ‘*One Site- Specific Binding*’ model (Y = B_max_ * X/(K_D_ + X) where X is the ligand concentration, Y is the fluorescence intensity, B_max_ is the maximum specific binding and K_D_ is the equilibrium binding constant) in GraphPad Prism 7.04.

### Assessments of galectin-ligand interactions by ITC

ITC experiments were performed at 25 °C using a MicroCal iTC_200_ microcalorimeter (Malvern Analytical Ltd, Malvern, UK) with 200 µl cell capacity and 40 µl injection syringe volume. All galectins were used at 100 µM in PBS and the binding ligands were prepared in identical buffers. LacNAc at 2 mM (for galectin-3), 20 mM (for galectin-2) or 100 mM (for full-length and truncated galectin-4); lactose at 30 mM (for galectin-2, -4 and truncated galectin-4) or 4 mM (for galectin-3); and galactose at 20 mM (for galectin-3) or 200 mM (for galectin-2, -4 and truncated galectin-4) were used in the binding titrations. F3 was titrated into galectin-3 at concentration of 20 mM. Ligand-to-buffer and buffer-to-galectin titrations were performed as controls. Ligands were titrated in 19 injections of 2 µl aliquots with 120 s spacing between injections to allow baseline recovery. The ITC titration data were analysed using MicroCal Data Analysis for iTC200 which is an Origin 7.0-based software programme. A curve fit using the ‘One *set of sites*’ interaction model was used to determine the association constant (K_A_), stoichiometry of binding (N) and enthalpy change (∆H) for all the interactions of simple sugars with the galectins except F3, where a logistic fit was used due to the nature of F3 as a mixture of heterogeneous polysaccharides.

## Supplementary information

Supplement figure
