# Supplementary material for: Intrinsic tryptophan fluorescence spectroscopy reliably determines galectin-ligand interactions
Source: Sci Rep. 2019 Aug 14;9:11851. doi: 10.1038/s41598-019-47658-8 (PMC6694196; doi:10.1038/s41598-019-47658-8)
Supplement: Supplementary file 1 — Supplement figure [file 41598_2019_47658_MOESM1_ESM.docx]

**Intrinsic tryptophan fluorescence spectroscopy reliably determines galectin-ligand interactions**

Paulina Sindrewicz^1^*, Xiaoxin Li^1^*, Edwin A Yates^2^, Jeremy E Turnbull^2^, Lu-Yun Lian^2^‡, Lu-Gang Yu^1^‡

^1^Department of Cellular and Molecular Physiology, Institute of translational Medicine; ^2^Department of Biochemistry, Institute of Integrative Biology, University of Liverpool, Liverpool L69 3GE, UK


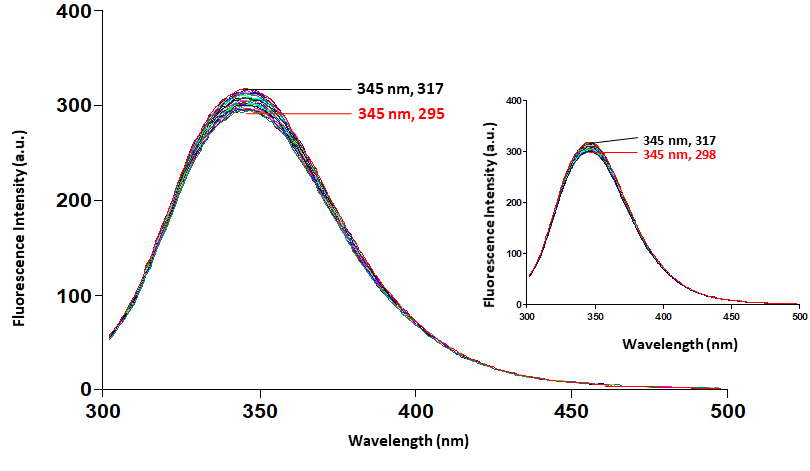


**Figure S1:** **BSA tryptophan fluorescence profile in the absence and presence of lactose.** BSA (5 µM) fluorescence intensity with increased concentrations of lactose was recorded by fluorescence spectroscopy. Numbers indicate the maximum fluorescence wavelength and corresponding fluorescence intensity for the first (no lactose, black) and last (6 mM, red) titration traces. Insert shows buffer control titrations.
